# In vitro evaluation of 4,4′-trimethylenedipyridinium and 4,4′-trimethylenedipiperidinium-based polycationic polymers against *Acanthamoeba hatchetti*

**DOI:** 10.1007/s00436-025-08537-6

**Published:** 2025-08-20

**Authors:** Tomas Rimkus, Stephan Reichl

**Affiliations:** 1 Institute of Pharmaceutical Technology and Biopharmaceutics, Technische Universität Braunschweig, Brunswick, Germany; 2 Center of Pharmaceutical Engineering, Technische Universität Braunschweig, Brunswick, Germany

**Keywords:** *Acanthamoeba*, Polycationic, Polymers, Preservatives, Trimethylenedipyridine, Trimethylenedipiperidine

## Abstract

Corneal exposure to trophozoites of *Acanthamoeba *spp. may lead to *Acanthamoeba* keratitis (AK)—a rare, but sight-threatening disease—with a risk of recurrence due to residual stromal cysts. With polyhexanide (PHMB) and chlorhexidine (CHX) often constituting the standard regimen of therapy, polymeric compounds for the treatment of AK have shifted into the focus of research. In this study, the effectiveness of four 4,4′-trimethylenedipyridinium (TMDPy), 4,4′-trimethylenedipiperidinium (TMDPi)-based polymers, and polyquaternium-1 on *Acanthamoeba* trophozoites and cysts has been evaluated and their interactions with cells characterized. A total eradication assay was performed to assess the efficacy of the investigated compounds, while its effects on host cells and the barrier integrity of epithelial cell layers were evaluated via MTT assays and the relative reduction of transepithelial electrical resistance (TEER). The 4,4′-trimethylenedipyridinium and 4,4′-trimethylenedipiperidinium-based compounds exhibited a high efficacy against trophozoites (< 20 µg/mL), while the cysticidal activity proved to be considerably lower (< 500 µg/mL). The detrimental effect on viability of host cells was time-dependent, while a near total reduction of TEER was observed within the first 15 min of exposure, leading to the conclusion that this class of polymers may not be adequate for therapeutic purposes, but possibly find use as preservatives for contact lens storage solutions.

## Introduction

*Acanthamoeba *spp. are a type of single-celled eukaryotes commonly found in the human environment, i.e., lakes, ponds, soil, and dirt, but also in contact lens storage solutions. The active stage—trophozoites—typically feed on bacteria but can cause a type of infectious keratitis called AK or—mostly in immunocompromised patients (Salameh et al. 2015; Damhorst et al. 2022)—amoebic encephalitis. The dormant, double-walled cyst stage, however, enables the organism to survive under harsh environmental conditions and withstand therapeutic intervention, facilitating its persistence in infected host tissue such as the human corneal stroma and possible recurrence of active infection following excystment. Thus, an intense therapeutic regimen over several months is often required.

With the incidence of reported cases rising significantly in North America, the UK, and other parts of Europe—predominantly among contact lens wearers (Lacerda and Lira 2021; List et al. 2021; Li and Shekhawat 2022)—the need for improved treatment options is evident (Carnt et al. 2018; Randag et al. 2019; Scruggs et al. 2019). It is important to note, though, that contributing factors might be the increasing knowledge of differential diagnostics and the rising awareness of AK in general, leading to earlier diagnosis, onset of treatment, and an improved outcome for the patient (Li and Shekhawat 2022).

Despite the emergence of new—in the context of AK—compounds with anti-acanthamoebic activity against trophozoites and cysts in vitro such as voriconazole (Schuster et al. 2006) among other azole-antimycotics (Stevens and Willaert 1980; Schuster 1993), miltefosine (Schuster et al. 2006), oxasqualenoids (Lorenzo-Morales et al. 2019), derivatives of ursolic acid (Sifaoui et al. 2019), amphotericin B (Taravaud et al. 2017), alkylphosphocholines, and quaternary ammonium compounds (Mooney et al. 2020), in vivo data based on animal studies or a clinical setting of AK with human subjects is comparatively scarce. There, several combinations of (potential) active pharmaceutical ingredients for therapy of AK and their curative outcomes have been described. Among them are voriconazole (Hirabayashi et al. 2019; Papa et al. 2020; Avdagic et al. 2021), miltefosine (Hirabayashi et al. 2019; Avdagic et al. 2021), hexamidine, propamidine isethionate, CHX, and PHMB (Papa et al. 2020; Dart et al. 2024). Of those, only propamidine and PHMB have been investigated in phase 3 clinical trials of AK (Orphanet: Research and trials n.d.; Dart et al. 2024), and as a singular compound, PHMB still appears to be the most effective in its therapy.

PHMB and CHX represent cationic, membrane-acting agents (Khunkitti et al. 1997) that are broadly used for the treatment of AK, often in conjunction with the debridement of the corneal epithelium (Sun et al. 2006; Li and Shekhawat 2022), ensuring sufficient permeation into the corneal stroma, where the amoeba persist. This led to further investigations examining the amoebicidal activity of a range of cationic compounds, among them oligomers, polymers, and dendrimers (Zanetti et al. 1995; Heredero-Bermejo et al. 2013; Shing et al. 2021). In this study, four polycationic, polymeric compounds with a 4,4′-trimethylenedipyridinium/dipiperidinium scaffold and alkyl linkers of varying length (C_8_/C_10_) have been synthesized and their efficacy against *A. hatchetti* (2HH strain) assessed, employing a total eradication assay derived from Gatti et al. (1998). The results thereof were then compared to those of polyquaternium-1 (PQ1), a polycationic polymer already used in contact lens storing solutions and other ophthalmic formulations. Here, the morphology, growth, and disintegration of *Acanthamoeba* trophozoites and cysts were observed over a period of 14 days after the initial exposure to anti-amoebic agents, as *Acanthamoeba* have been shown to recover from the initial damage done by anti-amoebic compounds and therefore entail the risk of overestimation of anti-amoebic activity employing staining techniques or spectroscopic methods with an earlier endpoint (Shi et al. 2019). The results obtained from this study demonstrate the high efficacy of polycationic polymers against trophozoites, but indicate an insufficient activity against cysts, while proving a lack of specificity by negatively impacting the viability and barrier integrity of epithelial cells.

## Methods

### Polycationic compounds

Polyquaternium-1 (PQ1) was kindly provided by ChiroBlock^®^ (Wolfen, Germany).

#### Syntheses of TMDPyC_8_ and TMDPyC_10_

1.002 g (5.05 mmol, 1.0 eq.) of 4,4-trimethylenedipyridine (TCI, Chuo, Japan) was solubilized in 20 mL acetone. 1.022 mL/1.248 mL (5.55 mmol, 1.1 eq.) of 1,8-dibromooctane (Alfa Aesar, Ward Hill, USA)/1,10-dibromodecane (TCI, Chuo, Japan) was added, and the mixture was heated to reflux for 0.5 h/0.75 h. Then, 0.422 mL (3.04 mmol, 0.6 eq.) of triethylamine (Alfa Aesar, Ward Hill, USA) was added, and the mixture was heated for 5 more hours to reflux. The resulting precipitate was filtered, rinsed with acetone, and dried under vacuum. TMDPyC_8_ and TMDPyC_10_ had a yield of approximately 43.63% and 37.79%, respectively.

The following shifts (ppm) were observed for TMDPyC_8_: 1.167 (TEA CH_3_, t, 3 H), 1.22–1.35 (CH_2_, m, 8 H), 1.90 (CH_2_, m, 4 H), 2.12 (CH_2_, m, 2 H), 2.93–3.02 (CH_2_, m, 4 H), 3.233 (TEA CH_2_, 2H), 4.51–4.63 (CH_2_, m, 4H), 8.02–8.11 (Ar–H, m, 4 H), 8.95–9.08 (Ar–H, m, 4H).

The following shifts (ppm) were observed for TMDPyC_10_: 1.172 (TEA CH_3_, t, 3 H), 1.20–1.41 (CH_2_, m, 10 H), 1.83–1.97 (CH_2_, m, 4 H), 2.08–2.17 (CH_2_, m, 2 H), 2.90–3.03 (CH_2_, m, 4 H), 3.246 (TEA CH_2_, qt, 2 H), 4.52–4.65 (CH_2_, m, 4H), 8.03–8.14 (Ar–H, m, 4 H), 8.90–9.12 (Ar–H, m, 4H).

#### Syntheses of TMDPiC_8_ and TMDPiC_10_

2.234 mL (8.40 mmol, 1.0 eq.) of 1,3-bis-(1-methylpiperidin-4-yl)-propane (Merck, Darmstadt, Germany) was solubilized in 40 mL of acetone. 1.702/2.076 mL (9.24 mmol, 1.1 eq.) of 1,8-dibromooctane/1,10-dibromodecane was added, and the mixture was heated to reflux for 15 h. 0.698 mL (5.04 mmol, 0.6 eq.) of triethylamine was added, and the mixture was heated for 5 more hours to reflux. The resulting precipitate was filtered, rinsed with acetone, and dried under vacuum. TMDPiC_8_ and TMDPiC_10_ had a yield of 50.66% and 78.22%, respectively.

The following shifts (ppm) were observed for TMDPiC_8_: 1.22–1.40 (CH, CH_2_, m, 16 H), 1.45–1.65 (m, 8 H), 1.68–1.82 (CH, CH_2_, m, 6 H), 3.00 (CH_3_, s, 3H), 3.04 (CH_3_, s, 3H), 3.21–3.40 (CH, CH_2_, m, 6 H), 3.40–3.50 (CH, m, 4 H).

**Fig. 1:**
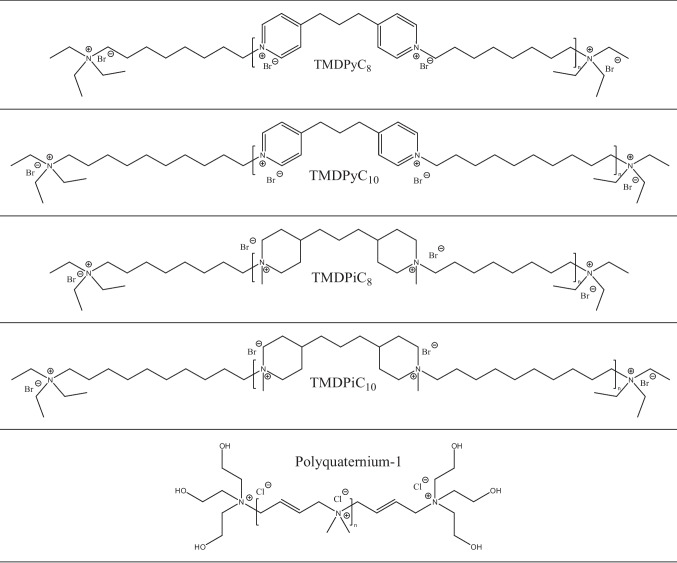
Structural formulae of the four synthesized, polycationic polymers with a 4,4′-trimethylenedipyridinium or 4,4′-trimethylenedipiperidinium scaffold and varying lengths of alkyl linkers (C_8_–C_10_) and polyquaternium-1

The following shifts (ppm) were observed for TMDPiC_10_: 1.32–1.47 (CH, CH_2_, m, 20 H), 1.60–1.77 (m, 8 H), 1.79–1.96 (CH, CH_2_, m, 6 H), 3.05 (CH_3_, s, 3H), 3.08 (CH_3_, s, 3H), 3.32–3.40 (CH, CH_2_, m, 6 H), 3.40–3.50 (CH, dd, 4 H). The structural formulae of all investigated compounds are illustrated in Fig. 1.

### *Acanthamoeba* culture

#### Trophozoites

Pathogenic *A. hatchetti* (2HH strain) trophozoites, kindly provided by the research group of Prof. Julia Walochnik (Vienna, Austria), were cultured in PYG medium (20.0 g proteose peptone, 20.0 g yeast extract, 18.0 g glucose, 8.0 mL 0.05 M CaCl_2_, 10 mL 0.4 M MgSO_4_ × 7H_2_O, 10 mL 0.25 M Na_2_HPO_4_ × 7H_2_O, 10 mL 0.25 M KH_2_PO_4_, 1.0 g sodium citrate, 10 mL 0.005 M Fe(NH_4_)_2_(SO_4_)_2_ (all from Carl Roth, Karlsruhe, Germany) in 1000 mL of distilled water) in an incubator at 30.0 °C and passaged weekly.

#### Cysts

Trophozoites cultivated as described above were centrifuged briefly; the PYG was replaced with Neff’s Encystation Medium (NEM) according to Kilvington and Lam (2013) containing 0.1 M KCl, 0.001 M NaHCO_3_, 0.008 M MgSO_4_ × 7H_2_O, 0.02 M Tris, 0.0004 M CaCl_2_, and 25 µL of phenol red-sodium solution (1.5% w/w in H_2_O) (all from Carl Roth) in 1000 mL of distilled water with the pH adjusted to 8.9 ± 0.1 and incubated at room temperature for 10–14 days until near complete encystation was observed. The cysts were subsequently washed with and stored in Page’s Amoeba Saline (PAS) containing 0.142 g Na_2_HPO_4_, 0.136 g KH_2_PO_4_, 0.004 g MgSO_4_ × 7H_2_O, 0.004 g CaCl_2_ × 2H_2_O, and 0.120 g NaCl (all from Carl Roth, Karlsruhe, Germany) in 1000 mL of distilled water at 4–8 °C until further usage.

### Amoebicidal assays

#### Determination of the minimal trophicidal concentration (MTC)

Confluent trophozoites were harvested after detachment on ice, and the suspension in PYG was centrifuged briefly. Then, the PYG medium was replaced with PAS, and the final suspension was adjusted to a density of 5 × 10^5^ cells/mL via hemocytometer (Neubauer type). Fifty microliters of this suspension was put into each well of a 96-well plate and left incubating at 30 °C for 30 min, facilitating attachment of the amoeba, whereupon 50 µL of the respective dilution (PAS for the controls) of the investigated substance was added. The plates were subsequently incubated at 30 °C for 2 h, after which all medium was gently aspirated and replaced with PYG. Finally, the plates were incubated for further 14 days, and on days 2, 4, 7, and 14, checked for growth, morphology of trophozoites, or disintegration. The concentration at which the absence of growth, morphologically intact cells, or total disintegration of trophozoites was observed was deemed the MTC, respectively. All experiments were performed twice using sextuples.

#### Determination of the minimal cysticidal concentration (MCC)

Stored cysts as described above were adjusted to a final density of 5 × 10^5^ cells/mL in PAS via hemocytometer (Neubauer type) and processed in the same manner as for the determination of the MTC. Additionally, each well was checked for excystation. The concentration at which absence of growth or no encystment was observed was deemed the MCC, respectively. All experiments were performed twice using sextuples.

### Cell culture

#### HCE-T

SV-40 immortalized human corneal epithelial cells (HCE-T, RCB Cat# RCB2280, RRID:CVCL_1272) (Araki-Sasaki et al. 1995) were purchased from RIKEN Cell Bank (Tsukuba, Japan) and cultivated in keratinocyte growth medium KGM (Lonza Bioscience, Basel, Switzerland) spiked with 700 µL of CaCl_2_ (27.75 mg/mL, Acros Organics, Geel, Belgium) in an incubator at 37 °C, 5% CO_2_, and 100% RH. They were fed three times per week and passaged weekly.

#### MDCK1

Madin-Darby canine kidney cells (MDCK1, ECACC Cat# 00062106, RRID:CVCL_0592), obtained from the European Collection of Authenticated Cell Cultures (ECACC, Salisbury, UK), were cultivated in MDCK medium containing 500 mL MEM eagle’s medium without l-glutamine, 5.68 mL l-glutamine 200 mM (both from PAN-Biotech, Aidenbach, Germany), 56.8 mL fetal bovine serum, and 5.68 mL antibiotic and antimycotic mixture 100 × (both from Sigma-Aldrich/Merck, Darmstadt, Germany) in an incubator at 37 °C, 5% CO_2_, and 100% RH. They were fed three times per week and passaged weekly.

#### Viability testing (MTT)

The impact of the investigated compounds on the viability of HCE-T cells upon exposure was assessed via an MTT assay. Briefly, 96-well plates were seeded with 2.5 × 10^4^ cells/well in KGM medium and incubated for 48 h at 37 °C, 5% CO_2_, and a RH of 100% until confluence was reached. The medium was gently aspirated and replaced with 100 µL of a solution of Krebs Ringer Buffer (0.13 g CaCl_2_ × 2H_2_O, 0.55 g d-glucose monohydrate, 1.79 g HEPES PUFFERAN^®^, 3.40 g NaCl, 1.05 g NaHCO_3_, 0.07 g NaH_2_PO_4_ × H_2_O (all from Carl Roth), 0.2 g KCl, and 0.1 g MgSO_4_ × 7H_2_O (both from Acros Organics) in 500 mL of distilled water, with the pH adjusted to 7.40 with 3 M NaOH (Carl Roth)), containing concentrations of compounds ranging from 390.6 to 6.104 µg/mL. The plates were then incubated for 30 to 60 min at the same conditions, after which the compound solutions were aspirated and replaced with 100 µL of MTT reagent (0.5 g MTT (Merck) in 10 mL distilled water) in KGM. After 60 min of further incubation, the medium containing MTT was aspirated, and 100 µL/well of lysis reagent was added, containing 1.365 g sodium dodecyl sulfate (Carl Roth), 452.7 g isopropanol, 1.7% HCl (37%) (both from Fisher Scientific), and 44.09 g double-distilled water. The plates were sealed from light and put on a shaker at 150 rpm for 2 h before measuring the absorption at 570 nm with a Tecan Infinite^®^ 200 Pro. Controls consisted of wells treated with KRB for 30 to 60 min of initial incubation. All experiments were performed twice using triplicates to sextuples.

#### Assessment of the relative reduction of the transepithelial electrical resistance (TEER)

With the TEER being a surrogate for barrier integrity of epithelial cell layers, the relative reduction of TEER after exposure of MDCK1 cells to the respective compounds over a period of 4 h was measured, as MDCK1 cells exhibit a high and stable TEER under normal conditions. Briefly, 10^5^ MDCK1 cells in MDCK medium were seeded into 12-well ThinCert^®^ plates (Greiner Bio-One, Kremsmünster, Austria) and incubated for 6 days, being fed on days 3, 5, and 6. Subsequently, the medium was aspirated and replaced with 600 µL of KRB and incubated for further 30 min to equilibrate. Then, the TEER was measured with an STX electrode. The initial TEER ranged from 4500 to 6500 Ω × cm^2^ for each insert. Finally, 66.6 µL of a solution containing 0.1% (w/v) compound was added to each well, resulting in a final concentration of 0.01% (w/v), and the TEER was measured after 5, 10, 15, 30, 45, 60, 90, 120, 150, 180, 210, and 240 min. For the control wells, 66 µL of KRB was added for the controls. All experiments were performed at least twice using duplicates to triplicates.

### Statistical analysis

Statistical analysis was performed employing the one-way ANOVA test using OriginPro 2019b (Northampton, USA). The tested level of significance was *P* < 0.05. The post hoc tests according to Bonferroni or Tukey were used when appropriate.

## Results

### Amoebicidal assays

#### Minimal trophicidal concentration

**Fig. 2:**
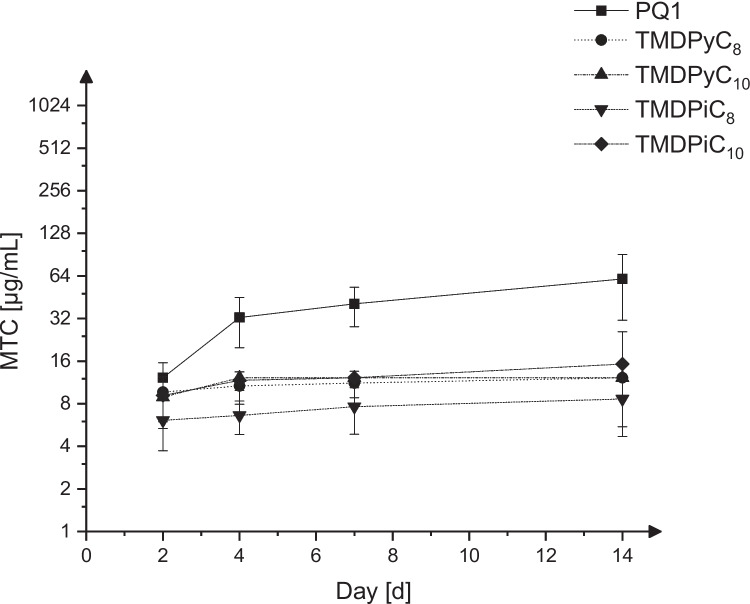
MTCs over a period of 14 days, mean ± SD, *n* = 6–12

The MTC curves for PQ1, TMDPyC_8_, TMDPyC_10_, TMDPiC_8_, and TMDPiC_10_ over a period of 14 days are displayed in Fig. 2. Only the final values after 14 days were considered for the comparative evaluation of efficacy. Here, TMDPiC_8_ has the lowest MTC (8.648 ± 3.144 µg/mL) and therefore the highest efficacy against 2HH trophozoites, followed by TMDPyC_8_ (12.21 µg/mL), TMDPyC_10_ (12.21 µg/mL), TMDPiC_10_ (15.26 ± 10.57 µg/mL), and PQ1 (61.04 ± 29.90 µg/mL).

Due to the low differences regarding their MTCs, no structure–activity relationship can be derived. It is notable, however, that all investigated trimethylene dipyridines/dipiperidines exhibit a significantly higher anti-acanthamoebic activity against trophozoites than PQ1 (*P* < 0.05) at days 4–14. Moreover, a time-dependent decrease in measured activity expressed as increasing MTCs could be seen for all compounds, suggesting that initially severely damaged trophozoites recuperated after a prolonged cultivation. This recuperation is also described in Shi et al. (2019), which concluded that the non-nutrient *Escherichia coli* growth assay is better suited to evaluate the in vitro efficacy of new compounds against *Acanthamoeba *spp., as spectroscopic assays such as the alamarBlue^TM^ assay might underestimate the recovery of amoeba and could therefore deliver misleading results (Shi et al. 2019).

#### Minimal cysticidal concentration

**Fig. 3:**
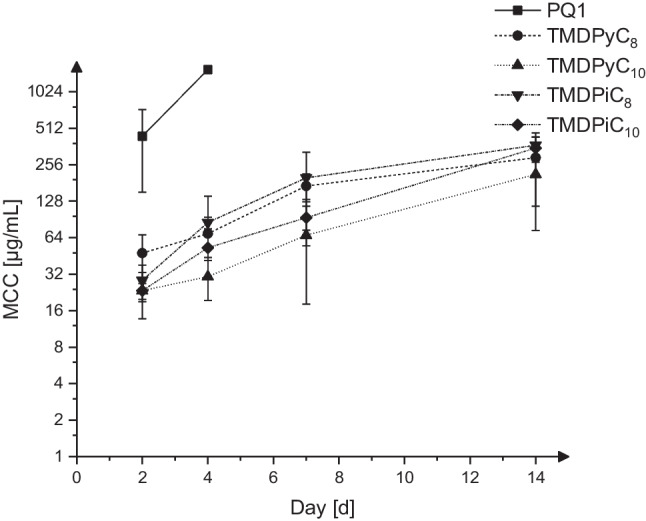
MCCs over a period of 14 days, mean ± SD, *n* = 5–12

The MCC_14d_s of all investigated compounds (Fig. 3) are substantially higher than the respective MTC_14d_s and range from 213.6 ± 140.1 for TMDPyC_10_ to 371.1 ± 61.76 for TMDPiC_8_, with TMDPyC_8_ and TMDPi_10_ being in between with MCCs of 293.0 ± 176.7 and 351.5 ± 82.35, respectively. Initially, TMDPi_8_ and TMDPi_10_ each exhibited *n* = 2 MCC_14d_s of > 390.6 µg/mL, which caused the test to be repeated with a wider range of concentrations, delivering no value > 390.6 µg/mL, suggesting that the previously measured MCC_14d_s of the two outliers lie between 781.3 and 390.6 µg/mL. Additionally, PQ1 reached the highest MCC_14d_ tested after 4 days of incubation, with subsequent measurements lying outside of the tested range (1563–1.526 µg/mL), suggesting a comparatively low activity against 2HH cysts as well as being significantly (*P* < 0.05) lower than that of all other investigated compounds after 2 to 4 days. An unsaturated scaffold and a longer alkyl linker appear to be linked to a higher activity against cysts, especially considering the respective MCCs at days 7 and 14, as can be derived from Table 1. Furthermore, the results indicate an early though asynchronous excystation of *Acanthamoeba*, as the MCCs are considerably higher than the MTCs at the same time points and rise quickly.

**Table 1:** MTC and MCC values for all investigated compounds over a period of 14 days, *–2 out of 12 samples showed an MCC > 390.6 μg/mL and were thus not included in the graphical or numerical representation, **–1 out of 6 samples showed an MCC > 1563 μg/mL and was not included in the graphical or numerical representation, mean ± SD, *n* = 5–12

Day [d]	MTC [μg/mL]				
	TMDPyC_8_	TMDPyC_10_	TMDPiC_8_	TMDPiC_10_	PQ1
2	9.665 ± 5.939	8.903 ± 3.555	6.104	9.157 ± 3.189	12.21
4	10.68 ± 2.762	12.21	6.613 ± 1.763	11.70 ± 1.763	32.55 ± 12.61
7	11.19 ± 2.377	12.21	7.631 ± 2.762	12.21	40.69 ± 12.61
14	12.21	12.21	8.648 ± 3.144	15.26 ± 10.57	61.04 ± 29.90
Day [d]	MCC [μg/mL]				
	TMDPyC_8_	TMDPyC_10_	TMDPiC_8_	TMDPiC_10_	PQ1
2	47.81 ± 19.79	23.39 ± 3.522	28.48 ± 9.505	23.40 ± 9.679	439.5 ± 288.1
4	69.18 ± 25.14	30.52 ± 11.04	85.45 ± 55.57	52.90 ± 22.89	1563^**^
7	170.9 ± 44.16	67.14 ± 48.96	199.4 ± 125.7	93.59 ± 38.72	> 1563
14	293.0 ± 176.7	213.6 ± 140.1	371.1 ± 61.76^*^	351.5 ± 82.35^*^	> 1563

### Reduction of cell viability, MTT

**Fig. 4:**
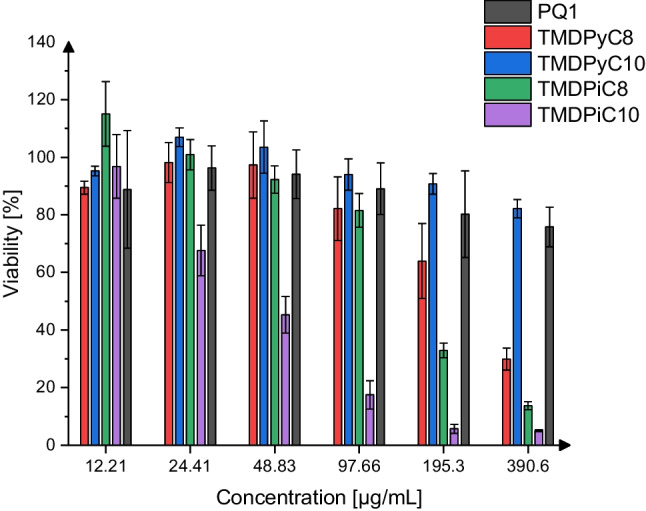
Relative viabilities of HCE-T cells after 30 min of incubation with the investigated substances, mean ± SD, *n* = 6–12

**Fig. 5:**
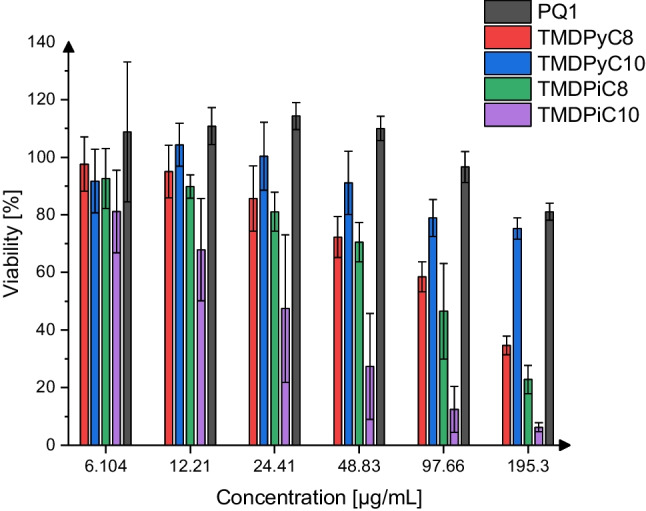
Relative viabilities of HCE-T cells after 60 min of incubation with the investigated substances, mean ± SD, *n* = 6–12

The effect of the polycationic compounds on the viability of human corneal epithelial cells after 30 to 60 min of incubation compared to KRB, which served as a control, is illustrated in Figs. 4 and 5. The concentrations were chosen to replicate the conditions employed in the MTC and MCC assays as well as to reflect the most likely range of concentrations these compounds would be applied in.

Time-dependent effects on cell viability were observed for all compounds and were most prominent for TMDPyC_8_ and TMDPiC_8_, which resulted in a viability of 63.93 ± 12.99% (30 min) and 34.60 ± 3.233% (60 min) and 32.87 ± 2.558% (30 min) and 22.80 ± 4.894% (60 min) at a concentration of 195.3 µg/mL, respectively. At a concentration of 12.21 µg/mL, none of the compounds led to a decrease in viability below 80% except for TMDPiC_10_, which exhibited the highest decrease in viability in the ranges of 390.3 to 24.41 µg/mL (30 min) and 195.3 to 6.104 µg/mL (60 min) of all compounds. At 24.41 µg/mL, the viability of the HCE-T cells was not reduced below 80% by TMDPyC_8_, TMDPyC_10_, TMDPiC_8_, and PQ1, indicating low cytotoxicity at highly trophicidal concentrations for the newly synthesized compounds. Overall, PQ1 and TMDPyC_10_ had the least detrimental impact on the viability of HCE-T cells, especially at higher concentrations (195.3–390.6 µg/mL, 30 min and 195.3 µg/mL, 60 min) and longer incubation times, followed by TMDPyC_8_. Compared to the benchmarking compound PQ1, after 30 min of exposition TMDPyC_8_ and TMDPyC_10_ diverged significantly (*P* < 0.05) at 390 µg/mL, while TMDPiC_8_ diverged significantly (*P* < 0.05) at concentrations of 12, 195, and 390 µg/mL and TMDPiC_10_ at all tested concentrations except for 6.104 µg/mL (*P* < 0.05). After 60 min of exposition, TMDPyC_10_ does not differ significantly from PQ1 regarding its impact on cell viability at any tested concentration, while TMDPyC_8_ and TMDPiC_8_ do so at concentrations of 24.41, 48.83, 97.66, and 195.3 µg/mL and TMDPiC_10_ at all investigated concentrations. This indicates a favorable tolerability of the pyridinium-based scaffolds over the piperidinium-based ones for this type of polymer while marking TMDPyC_10_ as the least toxic of the investigated compounds next to PQ1 towards HCE-T cells.

### Influence on integrity of epithelial barrier

**Fig. 6:**
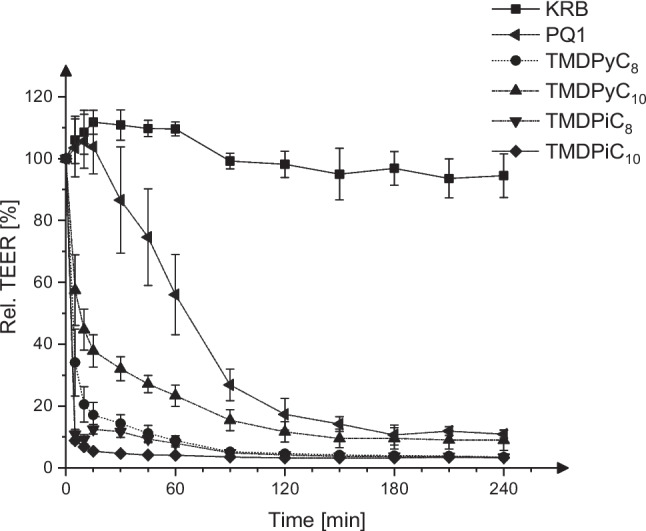
Relative TEER of a MDCK1 monolayer over a period of 4 h of incubation with the investigated substances, mean ± SD, *n* = 4–8

The results for the TEER measurements expressed as the relative reduction of TEER are illustrated in Fig. 6. The concentration of 0.01% was chosen to facilitate better comparability with previous studies (Deylen et al. 2018) on similar compounds and to simulate high exposure likely found in a real-world scenario considering trophicidal activities and the necessary safety margin-ensuring efficacy.

All investigated compounds exhibited a drastic reduction of rel. TEER over a span of 4 h at a concentration of 0.01% (w/v). After 5 min of incubation, TMDPyC_8_, TMDPiC_8_, and TMDPiC_10_ led to a decrease of TEER below 50% of the initial value. After 15 min, the TEER is reduced to < 20% of the initial value, which suggests a drastically negative impact on barrier integrity. The sharp drop in TEER is slightly less pronounced for TMDPyC_10_, although the thresholds of 50 and 20% rel. TEER were breached after 10 and 90 min, respectively. In comparison to the less potent PQ1, which leads to an initial increase of rel. TEER after the first 15 min, breaching the 50 and 20% rel. TEER thresholds after 90 and 120 min, respectively, all 4,4′-trimethylene dipyridinium/dipiperidinium-based compounds appear to be more harmful to the barrier integrity, especially considering short periods of exposition.

The reduction of TEER is most likely caused by extensive damage to and eventual lysis of cells, which is implicated by the membrane-acting properties of polycationic compounds (Carrier et al. 1985; Ikeda et al. 1990) and the reduction of viability after prolonged exposition as demonstrated previously.

## Discussion

Despite the advantages—such as high target specificity and increased bioavailability—small molecules can provide, cationic polymers remain a viable research option for compound research against *Acanthamoeba *spp., as these compounds often display a broad spectrum of efficacy against microbial threats such as bacteria (Al-Khalifa et al. 2017; Deylen et al. 2018; Mukherjee et al. 2020) and fungi (Deylen et al. 2018; Mukherjee et al. 2020), enabling their use as disinfectants, preservatives, or even therapeutic agents, given sufficient activity and tolerability. All four synthesized compounds exhibit higher anti-amoebic activities against *A. hatchetti* (2HH strain) trophozoites and cysts than PQ1 in terms of total eradication, illustrating the potential of different classes/scaffolds of polycationic compounds in the context of AK. However, a significant disadvantage may be the lack of specificity of these types of molecules, as the results obtained from this study indicate moderately to severely detrimental effects on host cells regarding viability and barrier integrity. Overall, these effects have been less evident for TMDPyC_10_, which showed similar characteristics as the reference compound PQ1 and could therefore be identified as a promising compound for further investigations. Furthermore, studies with highly similar structural scaffolds reported high bactericidal and mycocidal activities against *Enterococcus faecalis*, *E. coli*, *Pseudomonas aeruginosa*, *Staphylococcus aureus*, *Aspergillus brasiliensis*, and *Candida albicans* among others (Al-Khalifa et al. 2017; Deylen et al. 2018), suggesting a potential application as preservatives in ophthalmic formulations or even topical agents in therapy following thorough characterization. A combination with synergistic agents such as cellulose synthase inhibitors (Moon et al. 2015) might improve the efficacy of the polymers even further. But while the trophicidal potential of the investigated 4,4′-trimethylenedipyridine/dipiperidine polymers is high (< 20 µg/mL), the comparatively low activity against cysts suggests no improvement over PHMB or CHX, as a study conducted by Narasimham et al. (2002) determined that the MCCs range from 100 to 25 µg/mL for PHMB and from 100 to 1.56 µg/mL for CHX, depending on the isolate, although the testing conditions there involved a longer exposition of *Acanthamoeba* to drugs (48 h) and a lower inoculate than in this study. In conclusion, the results obtained in this study contribute to the knowledge base about the amoebicidal potential of (poly)cationic compounds, highlighting the trophicidal potential and moderate cysticidal activity of 4,4′-trimethylenedipyridinium and 4,4′-trimethylenedipiperidinium-based polymers. The superior amoebicidal activity of TMDPyC_10_ over PQ1 and comparably mild effect on HCE-T cell viability outlines TMDPyC_10_ as a potential preservative in ophthalmic formulations or more preferably contact lens storage solutions—considering its effects on the rel. TEER—following further characterization of its bactericidal and mycocidal activity.

## Data Availability

All data regarding spectra from syntheses and raw data used for Figs. 2–6 are available on request to the corresponding author.
